# Complete Mitochondrial Genomes of Two Species of the Genus *Loricaria* (Loricariidae: Loricariinae) and Its Phylogenetic Implications

**DOI:** 10.3390/ani16101537

**Published:** 2026-05-18

**Authors:** Xiaoli Zhang, Shuya Liu, Liping Chen

**Affiliations:** College of Environment and Life Health, Anhui University of Applied Technology, Hefei 230011, China

**Keywords:** genus *Loricaria*, Loricariidae, mitochondrial genome, phylogenetic analysis, taxonomic delimitations

## Abstract

*Loricaria*, the type genus of the armored catfish family Loricariidae, is a species-depauperate lineage in the subfamily Loricariinae, with only 18 valid species formally recognized to date. However, the extremely high morphological homogeneity across congeners of this genus has long posed great obstacles to accurate taxonomic delimitation when relying solely on phenotypic traits. In this study, we newly sequenced and fully annotated the complete mitochondrial genomes (mitogenomes) of *L. parnahybae* and *L. simillima*. The two assembled mitogenomes are 16,581 bp and 16,371 bp in length respectively, both carrying the conserved vertebrate mitochondrial gene set consisting of 37 functional units (22 transfer RNA genes, 13 protein-coding genes (PCGs), 2 ribosomal RNA genes) and 1 non-coding control region. Among Protein Control Genes, only the Cox1 gene uses GTG as the start codon, whereas the remaining 12 PCGs initiate with ATG. The observed stop codons include TAA, as well as the incomplete stop codons TA and T. We reconstructed phylogenetic trees using both maximum likelihood and Bayesian inference methods, based on a dataset covering mitogenome sequences of 22 Loricariidae species and 2 outgroup taxa. Phylogenetic analyses consistently supported the monophyly of Loricariinae, Hypostominae, and Hypoptopomatinae. These results clarify the evolutionary position of *L. simillima* and *L. parnahybae* within Loricariidae and will contribute to elucidating the complex phylogenetic relationships among Loricariidae species.

## Simple Summary

Loricariidae is noted for its complex taxonomic identification and the potential for hybridization among species. We conducted phylogenetic analyses by integrating our data with all available complete mitochondrial genomes from members of the family Loricariidae retrieved from GenBank, to rigorously assess the phylogenetic placement of *Loricaria simillima* and *L. parnahybae*. Our results support the division of the family Loricariidae into three main subfamilies, which are generally consistent with the subfamilies previously defined based on morphology. Our results also support the monophyly of Hypostominae and support the classification proposed by Lujan, which divides the subfamily into nine tribe-level clades. The two species belonging to the tribe Neoplecostomini are nested within the subfamily Hypoptopomatinae and are sister to the tribe Otothyrini. *L. simillima*, *L. parnahybae*, and *L. cataphracta* are three valid species within the genus *Loricaria*, which belongs to the subtribe Loricariini of the subfamily Loricariinae.

## 1. Introduction

Loricariidae is a strictly Neotropical catfish family, with more than 1000 valid species described to date [1,2,3]. This makes it the most species-rich family within the Siluriformes. A key characteristic of this family is the dorsoventrally flattened body covered with bony plates, with the mouth specialized into a prominent sucker-like structure. This oral modification enables adhesion to surfaces in aquatic environments. Among Loricariidae, there are currently six subfamilies: Delturinae, Lithogeninae, Loricariinae, Hypoptopomatinae, Hypostominae and Rhinelepinae [4]. This family is noted for its complex taxonomic identification and the potential for hybridization among species.

Whiptail catfish of the genus *Loricaria* serves as the type genus of the family Loricariidae (Siluriformes), originally described by Linnaeus in 1758 [1,5]. Species within this genus are distinguished from other genera by specific characteristics, including slender filamentous projections on the lips, a reduced number of bifid premaxillary teeth (typically 3–4 per side), and premaxillary teeth length approximately twice that of the lower jaw teeth [6,7]. The genus *Loricaria*, as diagnosed by Isbrücker [5], was confirmed as a monophyletic group by Rapp Py-Daniel [6] based on additional synapomorphies. Currently, Loricaria represents one of the smallest genera within the subfamily, comprising 22 valid species [3]. Its distribution spans the Amazon, Orinoco, Paraguay, and Paraná basins, as well as small coastal drainages in Guyana and Brazil [3,8]. Species of this genus typically inhabit sandy or muddy substrate environments ranging from island streams to large lowland rivers, floodplain lakes, and coastal areas [1,9]. Fish within this genus are relatively small in body size, reaching a maximum total length of only 29.5 cm, typically around 18 cm [1]. These species exhibit highly similar morphological characteristics, presenting significant taxonomic complexity [1,3].

*Loricaria simillima* Regan, 1904, is distributed in the extensive Amazon, Orinoco, and La Plata river basins across Venezuela, Ecuador, Peru, Brazil, Paraguay, and Argentina [1,10]. The maximum recorded length for adult specimens is slightly over 26 cm [8]. However, the catfish typically attains a length of 18 cm. The fish has a robust, stout body with an elongated, slender caudal peduncle. Its fins are large and triangular. *L. parnahybae* Steindachner, 1907, whose common name is Parnahyba Whiptail, is distributed in Rio Parnahyba, Victoria, Alto Parnaíba Municipality, Maranhão, Brazil [11]. These two species exhibit a highly similar morphology, with the exception that *L. parnahybae* has a longer snout than *L. simillima*. These two species primarily inhabit freshwater benthic environments such as river tributaries [1,11]. Some species of this family have been introduced into China through the aquarium trade and as ornamental fish, later spreading to natural waters in southern regions due to aquaculture escapes and intentional releases. They possess strong adaptability and can rapidly establish populations in eutrophic water bodies, threatening native ecosystems through competition for food and disruption of habitats [1,2].

Comparative analysis of whole mitogenome sequences of species from the genus *Loricaria* and other representative taxa within the family Loricariidae enables robust validation and elucidation of the phylogenetic position of genus *Loricaria* species in this family, and further provides novel insights into the evolutionary relationships across Loricariidae. To date, only one species within the genus *Loricaria* (*L. cataphracta*) has had its complete mitochondrial genome sequence reported [12]. Species delimitation within Loricaria remains contentious, and the geographic distributions of most species are still poorly understood—particularly in the case of the *L. cataphracta* species complex, whose taxonomy and distribution pose ongoing challenges [7,13,14]. To address these issues, we present here the first characterization of the complete mitogenomes of two representative Loricaria species, *L. simillima* and *L. parnahybae*, including detailed descriptions of their genomic structure and sequence features. Based on these newly generated sequences, we conducted phylogenetic analyses by integrating our data with all available complete mitochondrial genomes from members of the family Loricariidae retrieved from GenBank, to rigorously assess the phylogenetic placement of *L. simillima* and *L. parnahybae*.

## 2. Materials and Methods

### 2.1. Sample Collection and Genomic Sequencing

Biological specimens were collected on 1 April 2022, from an aquatic product market in Qinhuai District (32.005899° N, 118.841977° E), Nanjing City, Jiangsu Province. These samples were all ornamental fish introduced to China from Brazil, South America. The sample collection process complied with applicable laws and regulations in China. Immediately after collection, caudal fin tissues were immersed in absolute ethanol and preserved at −20 °C for long-term cryopreservation. Genomic DNA was isolated using the cetyltrimethylammonium bromide (CTAB) method [15]. DNA integrity was evaluated through 0.8% agarose gel electrophoresis, while purity was determined spectrophotometrically (A260/A280 ratio > 1.8). Fragmentation of genomic DNA was performed using a Covaris M220 ultrasonic device (Covaris Inc., Woburn, MA, USA) to generate random fragments of 300–500 bp. Libraries were prepared following Illumina^®^ standard protocols including end-repair, adenylation, and adapter ligation, followed by PCR amplification. Preliminary quantification utilized a Qubit 3.0 Fluorometer (Thermo Fisher Scientific, Waltham, MA, USA), with subsequent dilution [16]. Insert size distribution (mean: 350 ± 25 bp) was verified using a Qsep100 Bioanalyzer (BiOptic Inc., New Taipei, Taiwan, China). Effective library concentration (>3 nM threshold) was precisely measured via quantitative PCR (qPCR) using KAPA SYBR^®^ FAST kits (Roche Diagnostics, Indianapolis, IN, USA). After quality control (QC) verification, libraries were pooled equimolarly based on effective concentrations and target sequencing depth. Paired-end sequencing (2 × 150 bp) was performed on the Illumina HiSeq 4000 platform (Illumina Inc., San Diego, CA, USA), generating ≥10 Gb raw data per sample. Sequence quality was monitored using real-time analysis (RTA 3) software with Q30 scores > 85% across all samples.

### 2.2. Sequence Assembly

Raw sequencing data were subjected to quality control (QC) analysis to generate clean data. Filtering was performed using the NOVOPlasty 4.3.1 tool based on three criteria: (1) removal of reads containing adapters; (2) exclusion of reads with an N base content exceeding 10%; and (3) discarding of reads in which more than 50% of the bases had a quality value lower than 20 (Q20) [17]. The resulting clean reads were aligned to the reference genome using HISAT 2.2.1. Preliminary genome annotation was conducted using the MITOS web server [18]. Reference sequences with high similarity and close phylogenetic relationships were selected using BLAST 2.16.0, and the preliminary MITOS annotation results were validated against these references. Transfer RNA (tRNA) genes were systematically identified using the MITOS2 software (v2.1.10) for comprehensive mitochondrial genome analysis and tRNAscan-SE v2.0 for specialized tRNA detection [19].

### 2.3. Bioinformation Analysis

We estimated pairwise genetic distances of mitochondrial genomes across all species using MEGA X (version 11) [20]. Base composition asymmetry and synonymous codon usage bias were quantitatively assessed using MEGA software (version 11) [21]. Annotated mitochondrial genome sequences underwent rigorous compositional analysis with the following parameters: (1) whole-genome nucleotide frequencies (A%, T%, C%, G%), (2) strand-specific base distribution, and (3) relative synonymous codon usage (RSCU) indices calculated for all 13 protein-coding genes. This approach enables detection of mutational pressures and translational selection forces driving molecular evolution in mitochondrial DNA. Genomic strand asymmetry was evaluated through AT-skew and GC-skew metrics, computed according to established formulas: GC-skew = (G − C)/(G + C) and AT-skew = (A − T)/(A + T) [22]. To enable comprehensive structural and functional interpretation of the mitochondrial genome, a high-resolution circular map was systematically generated using the MitoFish web server v2026.04 [23].

### 2.4. Genetic Differentiation and Phylogenetic Analysis

We included the assembled mitochondrial genomes of *L. simillima* and *L. parnahybae*, along with 20 mitochondrial genomes of Loricariidae from GenBank, in the phylogenetic analysis [12,24,25,26,27,28,29,30]. *Bagroides melapterus* (family Bagridae) and *Clarias intermedius* (family Clariidae), which fall under the order Siluriformes as the studied Loricariidae taxa, were employed as outgroups. Phylogenetic analysis was conducted using mitochondrial protein-coding gene (CDS) sequences of the 24 species (Table 1). The mitochondrial genomes of these 24 species were processed using PhyloSuite v1.2.3 [31]. Multiple sequence alignments were initially conducted using MAFFT v7 [32] employing a codon-based alignment strategy. To further mitigate potential alignment artifacts and address issues specific to coding sequences, such as frameshift mutations or premature stop codons, all initial alignments were subsequently refined using the MACSE v2 program [33]. Following rigorous alignment and optimization protocols, the final concatenated dataset encompassed all 24 species under investigation. This dataset consisted of 13 conserved coding sequences (CDS), which were carefully screened to ensure homology and comparability across different species. Subsequently, the ModelFinder v2.6 plugin [34] was utilized to perform comprehensive optimal data partitioning and gene-specific model selection on the merged dataset.

Phylogenetic relationships were reconstructed using two complementary statistical frameworks: Bayesian Inference (BI) and Maximum Likelihood (ML). BI analyses were executed via the MrBayes 2.24 plugin [35], employing Markov Chain Monte Carlo (MCMC) algorithms to estimate posterior probabilities, whereas ML analyses were completed using the IQ-TREE 2.4.0 plugin [36], aiming to identify the tree topology that maximizes the likelihood of the observed data. Following evaluation by the model selection procedure, the best-fit substitution models were determined as follows: the GTR + F + I + G4 model, incorporating empirical base frequencies, proportion of invariant sites, and gamma-distributed rate heterogeneity, was adopted for BI analysis; in contrast, the slightly simplified yet sufficiently descriptive GTR + I + G model was applied for ML analysis. The integration of these two methods provided mutual corroboration, ensuring the high credibility of the final phylogenetic tree topology. Subsequently, visualization and annotation of the inferred phylogenetic tree were performed using the Interactive Tree Of Life (iTOL v5) online platform [37].

Genetic distances between Loricaria species and the other 21 species were estimated based on PCGs data of the 24 species using MEGA X [20].

## 3. Results

### 3.1. Basic Structure of Mitochondrial Genomes of L. simillima and L. parnahybae

**Figure 1:**
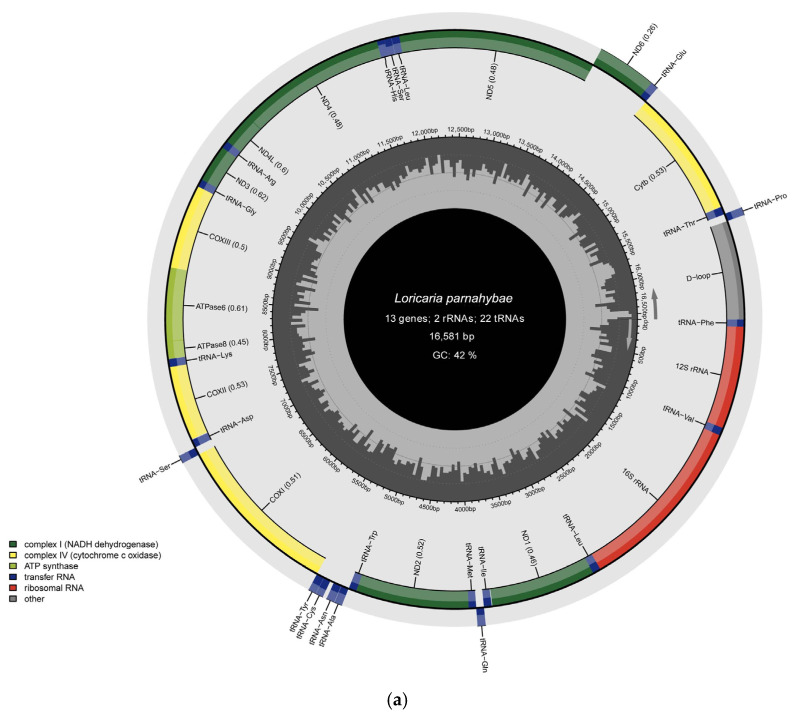

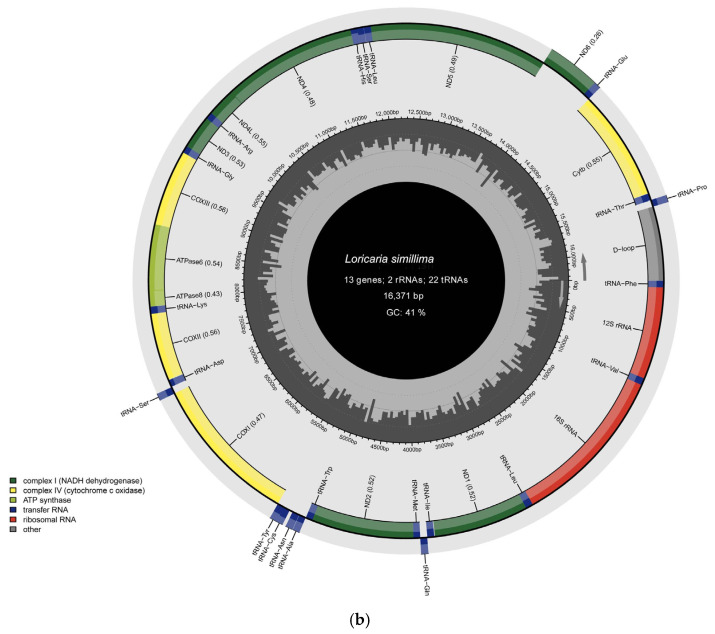
(**a**) Circular map of the mitochondrial genome of *Loricaria parnahybae*. (**b**) Circular map of the mitochondrial genome of *Loricaria simillima*.

The mitochondrial genome of *L. simillima* (GenBank: PQ524225) has a length of 16,371 bp and adopts the characteristic circular double-stranded conformation (Figure 1; Table 2). This genome comprises 37 functional genes and a control region spanning 761 bp. Gene mapping demonstrates that the Light (L) strand encodes nine genes: a single protein-coding gene (nad6) and eight tRNA genes (specifically tRNA^Gln^, tRNA^Ala^, tRNA^Asn^, tRNA^Cys^, tRNA^Tyr^, tRNA^Ser2^, tRNA^Glu^, tRNA^Pro^). Conversely, the heavy (H) strand harbors 28 genes, categorized into 12 protein-coding genes, 14 tRNA genes, and two rRNA genes. The non-coding control region is located on the Heavy (H) strand, with its start site at position 15667. Structural annotation further identifies 12 intergenic regions (1–35 bp), among which the longest (35 bp) flanks the tRNA^Cys^ and tRNA^Tyr^ loci. Additionally, five overlapping gene regions (1–10 bp) are detected, with the most extensive overlap (10 bp) occurring between the atp8 and atp6 genes.

In *L. parnahybae* (GenBank: PV243906), the mitochondrial genome extends to 16,581 bp and shares an identical circular double-stranded topology (Figure 1; Table 2). It likewise contains 37 functional genes, but features a significantly enlarged control region (915 bp) relative to *L. simillima*. Gene distribution exhibits striking conservation: the L strand carries nine genes (nad6 + eight tRNAs), while the H strand hosts 28 genes (12 protein-coding, 14 tRNAs, two rRNAs). The non-coding control region is located on the H strand, with its start site at position 15611. Structural scrutiny reveals 12 intergenic spacers of 1–33 bp, with the maximal interval again positioned between tRNA^Cys^ and tRNA^Tyr^. Five gene overlaps (1–10 bp) are also present, and the largest overlap similarly localizes to the atp8-atp6 junction, highlighting profound homology between these congeneric species.

### 3.2. Base Composition of Mitochondrial Genomes of L. simillima and L. parnahybae

Employing MEGA 11.0.13 software, we assessed the mitochondrial genome nucleotide composition of both *L. simillima* and *L. parnahybae* (Table 3). The total base frequencies revealed that *L. parnahybae* possessed A = 31.7%, C = 27.0%, T = 26.3%, and G = 15.1%, while *L. simillima* displayed A = 32.3%, C = 26.7%, T = 26.5%, and G = 14.5%. Both species exhibited significant A + T bias, with *L. simillima* accounting for 58.8% and *L. parnahybae* reaching 58.0%. Skew analysis further indicated compositional asymmetry, with positive AT-skew and negative GC-skew values (specifically, *L. parnahybae*: AT-skew = 0.093, GC-skew = −0.283; *L. simillima*: AT-skew = 0.098, GC-skew = −0.296). Focusing on protein-coding regions, *L. parnahybae* exhibited frequencies of A = 29.6%, C = 27.3%, T = 28.3%, and G = 14.8%, sustaining an A + T bias of 57.9%. Similarly, in *L. simillima*, these regions showed A = 30.2%, C = 26.6%, T = 28.9%, and G = 14.2%, corresponding to an A + T bias of 59.1%, thereby reinforcing the overall nucleotide preference pattern.

### 3.3. Protein-Coding Genes and Codon Usage

Both *L. simillima* and *L. parnahybae* demonstrated identical start codon patterns across their protein-coding genes (PCGs): twelve genes commenced with the canonical ATG codon, whereas cox1 uniquely employed GTG for initiation. In terms of stop codon utilization, uniformity prevailed among all thirteen PCGs in both species. Specifically, seven genes concluded with TAA, while nad2, cox2, cox3, nad3, and nad4 featured an incomplete T as the termination codon, and atp6 utilized an incomplete TA (Table 2).

**Figure 2:**
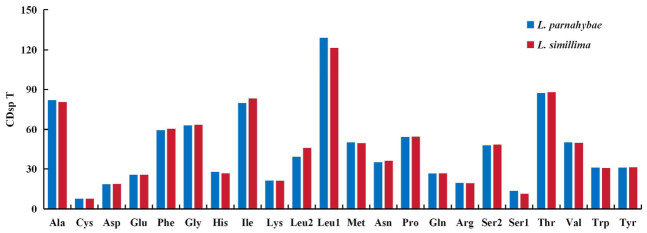
The codon utilization profile for the mitochondrial genomes of *L. simillima* and *L. parnahybae* is characterized by codon families depicted on the X-axis, while CDsp T on the Y-axis denotes codons per thousand codons.

**Figure 3:**
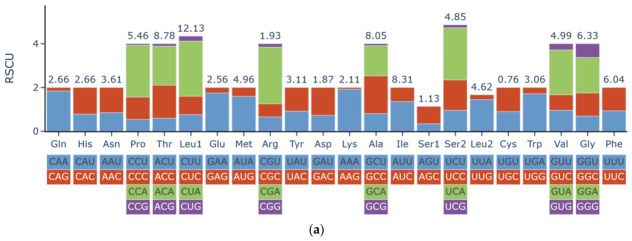

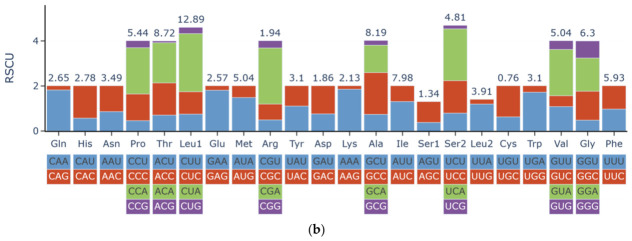
(**a**) Relative synonymous codon usage (RSCU) of the protein-coding genes (CDs) of *L. simillima* mitogenomes. (**b**) Relative synonymous codon usage (RSCU) of the protein-coding genes (CDs) of *L. parnahybae* mitogenomes.

**Figure 4:**
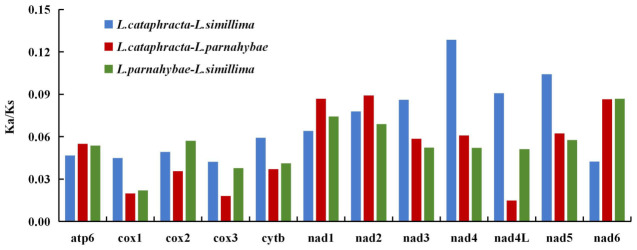
Comparative Ka/Ks ratio across 12 mitochondrial PCGs for three species of genus *Loricaria*. X-axis: Protein-coding gene identifiers; Y-axis: Ka/Ks ratio (nonsynonymous/synonymous substitution rate).

The total length of PCGs was 11,424 bp in *L. parnahybae* and 11,373 bp in *L. simillima*. The 13 PCGs comprised 3808 and 3791 codons, respectively, exhibiting high similarity in codon usage (as shown in Figure 2 and Figure 3). Leucine (Leu1) represented the predominant codon family in both species, exceeding 100 per thousand codons, likely associated with mitochondrial coding function. In contrast, Cysteine (Cys) was the least frequently used (as shown in Figure 2). This codon usage bias correlates closely with high A + T content, a characteristic feature of many animal mitochondrial genomes. Evaluation of Ka/Ks ratios revealed values less than 1 for all 12 PCGs, indicating functional stability and absence of strong adaptive evolution (Figure 4). Specifically, cox1, cox2, and cox3 exhibited the lowest ratios (0.019–0.045), suggesting stronger selective pressure, whereas the nad gene family showed relatively higher ratios (0.043–0.129), indicating weaker selective pressure.

### 3.4. Transfer and Ribosomal RNA Genes

The tRNA gene length ranges are consistent between *L. simillima* and *L. parnahybae*, both falling between 66 bp (tRNA^Cys^) and 75 bp (tRNA^Leu2^). The rRNA gene arrangement order is identical for the two species, with only minor length differences (1–2 bp) observed. In both *L. simillima* and *L. parnahybae*, the 12S rRNA (rrnS) gene is flanked by tRNA^Phe^ and tRNA^Val^, exhibiting near-identical lengths: 950 bp in *L. parnahybae* versus 948 bp in *L. simillima*. Similarly, the 16S rRNA (rrnL) gene resides between tRNA^Val^ and tRNA^Leu^ (annotated as tRNA^Leu2^), with lengths of 1,665 bp and 1,664 bp for *L. simillima* and *L. parnahybae*, respectively.

### 3.5. Genetic Distance and Phylogenetic Analysis

To explore sequence divergence among the three species of genus *Loricaria* and other species within the family Loricariidae, we analyzed the pairwise genetic distances based on 13 PCGs (Table 4). The PCGs of family Loricariidae showed significant genetic differentiation. Genetic distance between the *L. simillima* and *L. parnahybae* is about 11.0%. The genetic distance between *L. simillima* and *L. cataphracta* is about 5.8%. The genetic distance between the *L. parnahybae* and *L. cataphracta* is about 10.9%. The genetic distances between the genus *Loricaria* and those genera within the family Loricariidae are at least 15% (*Loricariichthys platymetopon*: 15.9–16.1%), with the majority exceeding 20%.

**Figure 5:**
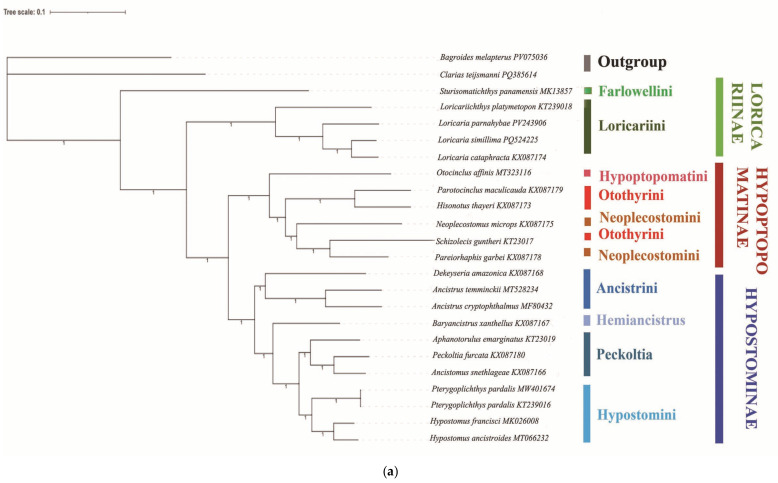

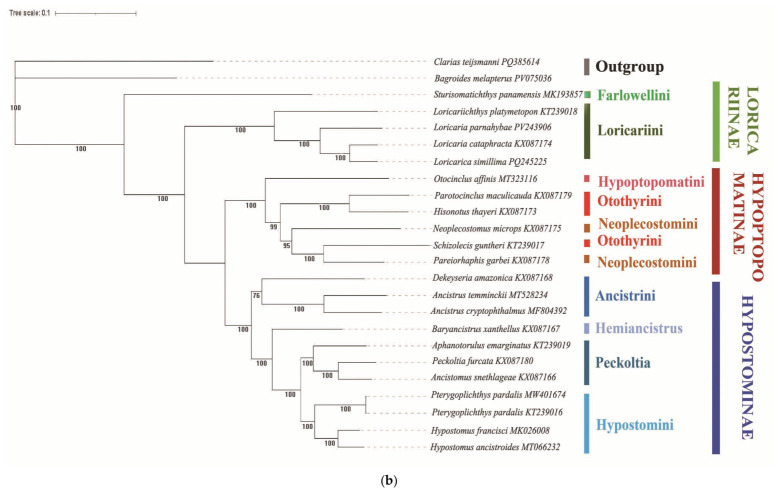
(**a**) Bayesian inference phylogeny of Loricariidae. Node labels indicate posterior probabilities (PP) from BI analysis, with values ≥ 0.95 denoting strong clade support. (**b**) Maximum likelihood (ML) phylogeny of Loricariidae. Nodal values indicate bootstrap support (BS) from ML analysis, with BS ≥ 70% denoting moderate and ≥95% strong clade stability.

Phylogenetic reconstructions employing both Bayesian inference (BI) and maximum likelihood (ML) methods robustly indicate that Loricariidae is divisible into three principal clades (Figure 5). These clades broadly align with the morphologically established subfamilies: Loricariinae (BI posterior probability = 1.0, ML bootstrap support = 100), Hypoptopomatinae (BI: 1.0, ML: 100), and Hypostominae (BI: 1.0, ML: 100). Substantial discrepancies, however, emerge in the topological arrangement and compositional integrity of these subfamilies within the phylogeny. Species belonging to the genus *Sturisomatichthys* of Loricariinae are excluded from the (Hypoptopomatinae + Hypostominae) Loricariinae) clade.

Both BI and ML analyses strongly support Loricariinae (BI: 1.0, ML: 100) as a monophyletic group and identify Loricariinae as the sister group to Hypoptopomatinae + Hypostominae. Within Loricariinae, the tribe Loricariini is supported as monophyletic (BI: 1.0, ML: 100). *Loricaria simillima* and *Loricaria parnahybae* clustered well with sequences of *Loricaria cataphracta* and *Loricariichthys platymetopon* obtained from GenBank, forming a well-supported clade within the Loricariini.

Both BI and ML analyses support the monophyly of the subfamily Hypoptopomatinae (BI: 1.00, ML: 100), and provide strong support for a sister-group relationship between Hypoptopomatinae and Hypostominae (BI: 1.0, ML: 100). However, Neoplecostominae and Otothyrini, which belong to Hypoptopomatinae, were found to be paraphyletic tribes. The tribe Hypoptopomatini is the sister group of Otothyrini + Neoplecostomini.

Hypostominae is well supported as a monophyletic subfamily (BI: 1.0, ML: 100). Both BI and ML analyses strongly support the division of genera within the subfamily Hypostominae into four distinct tribes (BI: 1.0, ML: 100). The tribe Ancistrini, composed of *Ancistrus cryptophthalmus*, *Ancistrus temminckii* and *Dekeyseria amazonica*, forms a monophyletic clade. The tribe Peckoltia, composed of *Ancistomus snethlageae*, *Aphanotorulus emarginatus* and *Peckoltia furcata*, forms a monophyletic clade. The tribe Hypostomini, composed of *Hypostomus ancistroides*, *Hypostomus francisci* and *Pterygoplichthys pardalis*, forms a monophyletic clade. The tribe Hemiancistrus, as analyzed in this study, contains only *Baryancistrus xanthellus*. Both BI and ML analyses supported a sister relationship between tribe Hypostomini and tribe Peckoltia. The tribe Hemiancistrus is the sister group of Hypostomini + Peckoltia. The tribe Ancistrini is the sister group of Hemiancistrus + Hypostomini + Peckoltia.

## 4. Discussion

The mitochondrial genomes of *L. simillima* and *L. parnahybae* exhibit parallels with those of other Loricariinae species regarding gene number and structural arrangement. Specifically, the mitochondrial DNA lengths for these species measure 16,581 base pairs and 16,371 base pairs, respectively, each containing 37 genes. This gene set comprises 22 transfer RNA genes, 13 protein-coding genes, 2 ribosomal RNA genes, and a single control region. The base composition characteristics of the mitochondrial genomes of the two species are highly consistent, and conform to the typical A + T bias rule of metazoan mitochondrial genomes [38,39,40,41]. Additionally, elevated AT content in DNA diminishes double helix stability due to AT base pairs forming only two hydrogen bonds, compared to the three hydrogen bonds in GC pairs [42]. Therefore, the high AT content of the mitochondrial genome may lead to a higher mutation rate, thereby potentially accelerating the evolutionary process of metazoans [39,43]. Indeed, compared to the AT-content observed in the invertebrate aquatic organisms of the Diaptomidae family (68.0–69.0%), the AT-content in *L. simillima* and *L. parnahybae* (58.0–59.0%), which are vertebrates, is significantly lower [44]. However, the AT-content of *L. simillima* and *L. parnahybae* is higher than the AT-content observed in the Cichlidae family (53.0–54.0%) [45].

The complete mitogenomes of *L. simillima* and *L. parnahybae* have 12 CDs using the ATG start codon; only cox1 uses GTG, a pattern also observed in other bony fishes [45,46]. Such incomplete termination codons (e.g., T or TA) have been documented in other fish mitochondrial genomes and are typically completed via post-transcriptional polyadenylation [40,47]. The codon usage patterns across other species within the Loricariini tribe display no notable variation. Mitochondrial genomes of *L. simillima* and *L. parnahybae* feature single-copy rrnL and rrnS genes without overlapping regions, in line with typical metazoan characteristics [48]. In addition, rRNA sequences of *L. simillima* and *L. parnahybae* are highly conserved and similar to those of other bony fish based on BLAST alignment [49]. For the entire set of 13 coding sequences (CDs) in both species, start codons resemble those in common bony fish, with GTG and ATG serving as the two most stable options; similarly, stop codons like TAA and the incomplete T are frequently observed in bony fish.

Previous studies on the evolutionary patterns of mitochondrial DNA have demonstrated that thermoregulation exerts a significant effect on the evolutionary mode of mitochondrial genomes, as evidenced by significantly lower Ka/Ks ratios of mitochondrial protein-coding genes in fish inhabiting cold climates than in tropical fish taxa [50,51]. In our study, all three analyzed genus *Loricaria* species are tropical taxa, with their Ka/Ks ratios ranging from 0.019 to 0.129, which are markedly higher than the Ka/Ks ratios reported for temperate and subtropical fish species in the existing literature. Furthermore, the Ka/Ks ratios of cox1, cox2 and cox3 in this study are significantly lower than those of NADH dehydrogenase gene family members, indicating that these three cytochrome c oxidase genes act as core evolutionary constraints shaping the evolutionary direction of the genus *Loricaria*.

Our reconstructed phylogenetic relationships support the monophyly of Loricariidae and the classification of its three subfamilies. This finding is generally consistent with previous morphologically based classifications of Loricariinae, Hypostominae, and Hypoptopomatinae. However, the phylogenetic relationships among species within the subfamily Loricariinae differ from those reported in prior phylogenetic studies.

For taxa in the subfamily Loricariinae, Covain et al. performed phylogenetic analyses based on 12S and 16S mitochondrial gene sequences obtained from 20 species representing 14 genera of this subfamily [52]. The results indicated that Loricariinae can be divided into two tribes: Harttiini, which only includes the genus *Harttia*, and Loricariini, which is further split into two subtribes, namely Loricariina and Farlowellina [52]. In the same study, the research team led by Covain also proposed a taxonomic hypothesis that Farlowellina, a clade affiliated with Loricariini, consists of four genera: *Farlowella*, *Lamontichthys*, *Sturisoma*, and *Sturisomatichthys*. This hypothesis was later supported by the research findings of Rodriguez et al. [53]. In recent years, Covain et al. carried out a new series of molecular phylogenetic studies on species within Loricariinae. In addition to mitochondrial genes, the nuclear marker f-rtn4 was incorporated into the analytical framework for these studies [54,55]. The updated results remain consistent with the previously established taxonomic framework that divides Loricariinae into two major clades, Harttiini and Loricariini. Specifically, Harttiini comprises three genera: Cteniloricaria, Harttia, and Harttiella; as a subtribe of Loricariini, Farlowellina contains six genera, namely *Aposturisoma*, *Farlowella*, *Lamontichthys*, *Pterosturisoma*, *Sturisoma*, and *Sturisomatichthys*. In this study, *Sturisomatichthys panamensis* is assigned to the subtribe Farlowellina, but it does not cluster with the tribe Loricariini within the subfamily Loricariinae. *S. panamensis* occupies a basal position in the phylogenetic tree, forming an unresolved clade. This may be attributed to the limited number of species within the subtribe Farlowellina included in the phylogenetic tree reconstruction, with only a single species represented.

Belonging to the subfamily Loricariinae, the genus *Loricaria* counts as one of the most species-depauperate genera in this subfamily, with only 22 confirmed valid species documented to date [1,3]. The high degree of morphological convergence among Loricaria species is the primary driver of its long-standing taxonomic confusion, and previous taxonomic studies on the genus have raised explicit controversies regarding the validity of several species: Liotta [56] considered *L. simillima* a synonym of *L. carinata*, whereas taxonomic revisions by Reis et al. [2], Ferraris [57], and Eschmeyer et al. [58] all supported *L. simillima* as an independent valid species, and assigned *L. carinata* as a synonym of *L. cataphracta*. In addition to morphological convergence, natural interspecific hybridization within the genus is also considered an important contributor to Loricaria taxonomic ambiguity. According to species delimitation conclusions for Loricaria proposed by Londoño-Burbano et al., a mitochondrial genetic distance exceeding 5% serves as the threshold for defining new species in this genus [14]. Genetic distances calculated based on complete mitochondrial genome sequences in this study showed that the interspecific genetic distance between *L. simillima* and *L. parnahybae* was approximately 5.8%, and the genetic distance between *L. parnahybae* and *L. cataphracta* was approximately 11.0%. Further alignment of the cox1 sequences of the two target species with all publicly available Loricaria cox1 sequences deposited in the NCBI database revealed that the genetic distances between both sequences and other congeneric species all exceeded 5% (see Supplementary Table S1). Since the present study was conducted only based on mitochondrial genome data, with no nuclear genetic markers applied to rule out the possibility of hybrid individuals, we can only confirm that the two complete mitogenome sequences generated in this study are authentic and reliable, but cannot directly verify the accuracy of the corresponding species identification. Further verification for *L. simillima* showed that the cox1 sequence of *L. simillima* obtained in this study shared over 99% sequence identity with reference *L. simillima* sequences from Brazilian localities in NCBI, which indirectly supports the validity of our *L. simillima* identification. Meanwhile, given the established taxonomic consensus that *L. carinata* is a synonym of *L. cataphracta*, the 5.8% genetic distance between *L. simillima* and *L. cataphracta* in this study (Table 4) also confirms that *L. simillima* and *L. carinata* are not heterotypic synonyms, consistent with prevailing taxonomic revision conclusions. In contrast, verification results for *L. parnahybae* showed that the cox1 sequence obtained in this study exhibited 11.4% genetic divergence from publicly available reference *L. parnahybae* cox1 sequences in NCBI, and its genetic divergence from all other congeneric species was also above 8%. This result indicates that the sample morphologically identified as *L. parnahybae* in this study either represents a case of morphological misidentification, or is a hybrid between two Loricaria species. Its exact taxonomic status remains to be unambiguously determined based on additional genetic data for the genus, coupled with systematic taxonomic assessments.

Based on a morphological study, Armbruster [59] considered Hypostominae to be a paraphyletic group. Lujan et al. [60] conducted a molecular phylogenetic reassessment of genus-level relationships within Hypostominae based on two mitochondrial and three nuclear gene loci. The study supported Hypostominae as monophyletic and that it can be divided into nine tribe-level clades among its genera (Chaetostoma, Ancistrini, Pseudancistrus, Lithoxus, “Pseudancistrus”, Acanthicus, Hemiancistrus, Hypostomini, Peckoltia). The phylogenetic relationships reconstructed in this study based on mitochondrial genome data from 13 protein-coding genes (PCGs) show that ten species belonging to the subfamily Hypostominae are clustered into four tribe-level clades: Ancistrini, Hemiancistrus, Hypostomini, and Peckoltia. Our results support the monophyly of Hypostominae and the classification proposed by Lujan, which divides the subfamily into nine tribe-level clades [60].

The subfamily Hypoptopomatinae constitutes a monophyletic clade containing roughly 85 small-bodied species [60]. Schaefer [61] was the first to include all recognized Hypoptopomatinae genera in a morphology-based phylogenetic analysis, resolving them into two tribes: Hypoptopomatini and Otothyrini with Neoplecostomus designated as the outgroup. The monophyly of these two tribes, along with their sister-group relationship when Neoplecostominae was excluded from analysis, was later validated by supplementary morphological data provided by Rapp Py-Daniel [6] and Schaefer [62]. Lujan et al. [60] subsequently revised the circumscription of Neoplecostominae, expanding its taxonomic scope to include Isbrueckerichthys, Kronichthys, Pareiorhaphis (which was classified under Hemipsilichthys at the time) and Pareiorhina. However, his analytical results also indicated that this newly expanded Neoplecostominae formed a paraphyletic group with respect to Hypoptopomatinae. Follow-up molecular phylogenetic studies yielded different findings: these works demonstrated that all genera historically assigned to Neoplecostominae form a monophyletic lineage nested within Hypoptopomatinae, with conflicting topologies placing this lineage either as the sister clade of Hypoptopomatini [63] or as the sister group of Otothyrini [60,64,65]. Our analytical results align with the latter topological pattern: the two sampled species belonging to tribe Neoplecostomini are nested within Hypoptopomatinae, and are recovered as the sister group to tribe Otothyrini.

Despite certain limitations, the present study generated two high-quality, publicly available complete mitogenome sequences of the genus Loricaria, providing novel baseline molecular data for resolving the phylogenetic relationships within the family Loricariidae. The limitations of this study are mainly reflected in two aspects: No nuclear genetic markers were supplemented for sample validation, so the possibility that the studied samples are congeneric hybrid individuals cannot be ruled out, leading to remaining uncertainty in the species identification of the sample initially morphologically assigned to *L. parnahybae* (notably, the cox1 sequence of *L. simillima* in this study shared over 99% sequence identity with reference *L. simillima* sequences deposited in NCBI, so its species identification is considered highly reliable). The phylogenetic framework of Loricariidae was constructed only based on mitochondrial genome markers. However, the mitochondrial genome is maternally inherited and can only reflect the matrilineal evolutionary history of taxa, which does not always fully match the true species-level evolutionary relationships. When events such as historical/recent introgressive hybridization or incomplete lineage sorting occur during the evolution of taxa, mitochondrial phylogenetic results are more prone to bias, which is the core reason for temporarily unresolved evolutionary relationships of some taxa in this study. The phylogenetic framework of Loricariidae was constructed solely based on mitochondrial genome markers. However, the mitochondrial genome is maternally inherited and can only reflect the matrilineal evolutionary history of taxa, which does not always fully match the true species-level evolutionary relationships. When events such as historical/recent introgressive hybridization or incomplete lineage sorting occur during the evolution of taxa, mitochondrial phylogenetic results are more prone to bias, which is the core reason for the low support values of certain phylogenetic nodes and the temporarily unresolved evolutionary relationships of some taxa in this study.

## 5. Conclusions

This study sequenced the complete mitochondrial genomes of two species within the genus *Loricaria* (Loricariidae). Based on gene arrangement patterns, codon usage characteristics, and conserved genetic variation features derived from these genomes, we conducted phylogenetic analyses to investigate interspecific relationships within the Loricariidae family. Our findings provide novel molecular genetic insights into the phylogenetic relationships among taxa within this family. However, the phylogenetic positions of several species remain unresolved in the analysis due to the absence of mitochondrial genome data for key closely related taxa. Therefore, obtaining more complete mitochondrial genomes across additional Loricariidae species will help establish a critical foundation for refining the phylogenetic framework of this family and elucidating its evolutionary origins and diversification mechanisms.

## Figures and Tables

**Table 1:** Details of species information employed in the phylogenetic reconstruction.

ID	Species	Original Source	Reference	Full
PQ524225	*Loricaria simillima*	aquatic product market in Nanjing, China	This study	16,371
PV243906	*Loricaria parnahybae*	aquatic product market in Nanjing, China	This study	16,581
MW401674.1	*Pterygoplichthys pardalis*	Xi’an breeding base, China	[24]	16,425
MT323116.1	*Otocinclus affinis*	Museum of Zhejiang Ocean University, Hangzhou, China	[25]	16,632
MT066232.1	*Hypostomus ancistroides*	Tietê river basin, near the municipality of Conchas, São Paulo, Brazil	[26]	16,826
MT528234.1	*Ancistrus temminckii*	Surin of South America	[27]	16,657
MK193857.1	*Sturisomatichthys panamensis*	Huadiwan Flowers and Birds Mart, in Guangzhou, China	[28]	16,497
MK026008.1	*Hypostomus francisci*	Abaeté River, a tributary of the São Francisco River, Minas Gerais, Brazil	[29]	16,541
MF804392.1	*Ancistrus cryptophthalmus*	Serra do Calca’rio, Goias State, Brazil	[30]	16,422
KX087180.1	*Peckoltia furcata*	Amazon basin, coastal basins of southeastern Brazil	[12]	16,313
KX087179.1	*Parotocinclus maculicauda*	Amazon basin, coastal basins of southeastern Brazil	[12]	16,202
KX087178.1	*Pareiorhaphis garbei*	Amazon basin, coastal basins of southeastern Brazil	[12]	16,269
KX087175.1	*Neoplecostomus microps*	Amazon basin, coastal basins of southeastern Brazil	[12]	16,477
KX087174.1	*Loricaria cataphracta*	Amazon basin, coastal basins of southeastern Brazil	[12]	16,409
KX087168.1	*Dekeyseria amazonica*	Amazon basin, coastal basins of southeastern Brazil	[12]	16,439
KX087167.1	*Baryancistrus xanthellus*	Amazon basin, coastal basins of southeastern Brazil	[12]	16,539
KX087166.1	*Ancistomus snethlageae*	Amazon basin, coastal basins of southeastern Brazil	[12]	16,333
KX087173.1	*Hisonotus thayeri*	Amazon basin, coastal basins of southeastern Brazil	[12]	16,167
KT239019.1	*Aphanotorulus emarginatus*	Amazon basin, coastal basins of southeastern Brazil	[12]	16,464
KT239018.1	*Loricariichthys platymetopon*	Amazon basin, coastal basins of southeastern Brazil	[12]	16,262
KT239017.1	*Schizolecis guntheri*	Amazon basin, coastal basins of southeastern Brazil	[12]	15,359
KT239016.1	*Pterygoplichthys pardalis*	Amazon basin, coastal basins of southeastern Brazil	[12]	16,520
PV075036.1	*Bagroides melapterus*	/	Unpublished	16,542
PQ385614.1	*Clarias teijsmanni*	/	Unpublished	16,514

**Table 2:** Genes organization and other information of the mitochondrial genomes in *L. simillima* and *L. parnahybae*.

Gene	*Loricaria parnahybae*							*Loricaria simillima*						
	Position		Size	Intergenic Nucleotides	Codon		Strand	Position		Size	Intergenic Nucleotides	Codon		Strand
	From	To			Start	Stop		From	To			Start	Stop	
*tRNA-Phe*	1	68	68	0			H	1	68	68	0			H
*12S RNA*	69	1018	950	0			H	69	1016	948	0			H
*tRNA-Val*	1019	1090	72	0			H	1017	1088	72	0			H
*16S RNA*	1091	2754	1664	0			H	1089	2753	1665	0			H
*tRNA-Leu2*	2755	2829	75	0			H	2754	2828	75	0			H
*ND1*	2830	3801	972	0	ATG	TAA	H	2829	3800	972	0	ATG	TAA	H
*tRNA-Ile*	3808	3879	72	6			H	3807	3878	72	6			H
*tRNA-Gln*	3880	3950	71	0			L	3880	3950	71	1			L
*tRNA-Met*	3950	4019	70	−1			H	3950	4019	70	−1			H
*ND2*	4020	5064	1045	0	ATG	T	H	4020	5064	1045	0	ATG	T	H
*tRNA-Trp*	5065	5135	71	0			H	5065	5135	71	0			H
*tRNA-Ala*	5138	5206	69	2			L	5138	5206	69	2			L
*tRNA-Asn*	5209	5281	73	2			L	5209	5281	73	2			L
*tRNA-Cys*	5317	5382	66	35			L	5315	5380	66	33			L
*tRNA-Tyr*	5384	5453	70	1			L	5381	5450	70	0			L
*COX1*	5455	7005	1551	1	GTG	TAA	H	5452	7002	1551	1	GTG	TAA	H
*tRNA-Ser2*	7006	7076	71	0			L	7003	7073	71	0			L
*tRNA-Asp*	7081	7154	74	4			H	7078	7150	73	4			H
*COX2*	7161	7851	691	6	ATG	T	H	7156	7846	691	5	ATG	T	H
*tRNA-Lys*	7852	7925	74	0			H	7847	7920	74	0			H
*ATPase8*	7927	8094	168	1	ATG	TAA	H	7922	8089	168	1	ATG	TAA	H
*ATPase6*	8085	8767	683	−10	ATG	TA	H	8080	8762	683	−10	ATG	TA	H
*COX3*	8768	9551	784	0	ATG	T	H	8763	9546	784	0	ATG	T	H
*tRNA-Gly*	9552	9623	72	0			H	9547	9618	72	0			H
*ND3*	9624	9972	349	0	ATG	T	H	9619	9967	349	0	ATG	T	H
*tRNA-Arg*	9973	10,042	70	0			H	9968	10,037	70	0			H
*ND4L*	10,043	10,339	297	0	ATG	TAA	H	10,038	10,334	297	0	ATG	TAA	H
*ND4*	10,333	11,713	1381	−7	ATG	T	H	10,328	11,708	1381	−7	ATG	T	H
*tRNA-His*	11,714	11,782	69	0			H	11,709	11,777	69	0			H
*tRNA-Ser1*	11,783	11,849	67	0			H	11,778	11,844	67	0			H
*tRNA-Leu1*	11,851	11,923	73	1			H	11,846	11,918	73	1			H
*ND5*	11,924	13,750	1827	0	ATG	TAA	H	11,919	13,745	1827	0	ATG	TAA	H
*ND6*	13,747	14,265	519	−4	ATG	TAA	L	13,742	14,260	519	−4	ATG	TAA	L
*tRNA-Glu*	14,266	14,333	68	0			L	14,261	14,328	68	0			L
*CYTB*	14,338	15,522	1185	4	ATG	TAA	H	14,333	15,466	1134	4	ATG	TAA	H
*tRNA-Thr*	15,526	15,598	73	3			H	15,470	15,542	73	3			H
*tRNA-Pro*	15,597	15,666	70	−2			L	15,541	15,610	70	−2			L

**Table 3:** Base composition of the *L. simillima* and *L. parnahybae* mitogenome.

Species	Regions	T (U)	C	A	G	AT (%)	GC (%)	AT Skew	GC Skew
*L. parnahybae*	Full genome	26.3	27.0	31.7	15.1	58.0	42.0	0.093	−0.283
	CDS (Coding sequences)	28.3	27.3	29.6	14.8	57.9	42.1	0.022	−0.297
*L. simillima*	Full genome	26.5	26.7	32.3	14.5	58.8	41.2	0.098	−0.296
	CDS	28.9	26.6	30.2	14.2	59.1	40.9	0.022	−0.303

**Table 4:** Pairwise genetic distance of 13 PCGs among 22 species within the family Loricariidae.

Species	*L. cataphracta*	*L. parnahybae*	*L. simillima*
*Ancistomus snethlageae*	0.211	0.212	0.209
*Ancistrus cryptophthalmus*	0.207	0.211	0.207
*Ancistrus temminckii*	0.221	0.223	0.217
*Aphanotorulus emarginatus*	0.214	0.216	0.214
*Baryancistrus xanthellus*	0.210	0.212	0.210
*Dekeyseria amazonica*	0.207	0.211	0.208
*Hisonotus thayeri*	0.226	0.226	0.225
*Hypostomus ancistroides*	0.206	0.208	0.208
*Hypostomus francisci*	0.209	0.211	0.210
***Loricaria cataphracta***	**/**	**0.109**	**0.058**
***Loricaria parnahybae***	**0.109**	**/**	**0.110**
***Loricaria simillima***	**0.058**	**0.110**	**/**
*Loricariichthys platymetopon*	0.161	0.159	0.161
*Neoplecostomus microps*	0.231	0.233	0.232
*Otocinclus affinis*	0.226	0.222	0.227
*Pareiorhaphis garbei*	0.226	0.227	0.227
*Parotocinclus maculicauda*	0.225	0.226	0.224
*Peckoltia furcata*	0.213	0.215	0.213
*Pterygoplichthys pardalis*	0.201	0.206	0.201
*Pterygoplichthys pardalis*	0.202	0.206	0.202
*Schizolecis guntheri*	0.241	0.243	0.240
*Sturisomatichthys panamensis*	0.231	0.233	0.232

Note: The content in bold refers to the genetic distances between the two species studied in this research and other congeneric species.

## Data Availability

The datasets presented in this study can be found in online repositories. The names of the repository/repositories and accession number(s) can be found below: https://www.ncbi.nlm.nih.gov/genbank/ PQ524225; https://www.ncbi.nlm.nih.gov/genbank/ PV243906. (These data can be accessed after 1 August 2026.)

## Supplementary Materials

The following supporting information can be downloaded at: https://www.mdpi.com/article/10.3390/ani16101537/s1, Table S1: Genetic distances between the two species in this study and other congeneric species based on *cox1* sequence data.
